# A comparison of the risk of cesarean section in gestational diabetes mellitus patients supplemented antenatally with vitamin D containing supplements versus placebo: A systematic review and meta-analysis of double-blinded randomized controlled trials

**DOI:** 10.4274/jtgga.galenos.2020.2019.0164

**Published:** 2020-09-03

**Authors:** Sumanta Saha, Sujata Saha

**Affiliations:** 1 National Institute for Research in Tuberculosis, India; 2 Mankar College, India

**Keywords:** Diabetes, gestational, vitamin D, cesarean section, fetal macrosomia, pre-eclampsia, premature birth, polyhydramnios

## Abstract

The aim of this study was to study the role of vitamin D containing supplements in the risk of cesarean section (CS), a common complication in gestational diabetes mellitus (GDM) patients. An additional objective was to assess the risk of developing pre-eclampsia, preterm delivery, macrosomia, and polyhydramnios in these participants. Various electronic databases were searched for double-blinded parallel-arm randomized controlled trials that reported the incidence of CS in adult, non-insulin treated GDM patients who received vitamin D and placebo in different treatment arms, respectively. Next, each eligible trial’s risk of bias was assessed, and the effects of the above interventions on the respective outcomes were compared meta-analytically across the trials. This review included five Iranian trials sourcing data from nearly 380 participants. The risk of bias in the trials was primarily low. In contrast to the placebo group, the risk of CS [risk ratio (RR): 0.61, p=0.002, 95% confidence interval (CI): 0.44,0.83; I^2^=0%, p-value of Cochrane’s Q: 0.373) and macrosomia (RR: 0.31, p=0.006, 95% CI: 0.13,0.72; I^2^=0%, p-value of Cochrane’s Q: 0.935] was less in the vitamin D supplemented group. The remaining outcomes did not differ between the intervention groups. The antenatal use of vitamin D containing supplements in non-insulin treated GDM patients might reduce the risk of CS and macrosomia.

## Introduction

Gestational diabetes mellitus (GDM) is a glucose intolerance to any degree occurring at the start of pregnancy or first recognized during gestation (1). It is diagnosed between 24-28 weeks of gestation using screening tests with a 50 gram and 1-hour glucose challenge test (1). It is classified as either A1GDM or A2GDM, depending on whether it is managed with dietary therapy or medication, respectively (1). The chief medication used to treat GDM if diet and exercise therapy fails is insulin (1). Glyburide and metformin, two oral hypoglycemic agents with the potential to cross the placenta, are also used to treat GDM frequently. However, such use of these medications is not approved by the U.S. Food and Drug Administration due to inadequate safety information (1,2). Unlike type 1 and type 2 diabetes, newer drugs such as sodium-glucose linked transporter 2 inhibitors, remain poorly studied in GDM patients (3,4,5).

GDM can cause both neonatal complications including macrosomia, neonatal hypoglycemia, shoulder dystocia, and hyperbilirubinemia and maternal complications (1,6). One of the chief maternal complications of GDM is cesarean section (CS), in which the fetus is delivered surgically by incising the abdomen and uterus of the parturient (1,7,8,9). The prevalence of CS is high in GDM patients (32-44%), and it is more common than in parturients with no glucose intolerance (7,10,11,12,13,14,15). The indication for CS is determined by the obstetric need of the GDM mother and includes indications such as pre-eclampsia, macrosomia, excessive fetal growth (fetal weight more than 4500 gm), and past obstetric history, for example previous history of childbirth by CS (7,8,16,17,18). CS increases the risk of wound hematoma, anesthetic complications, major puerperal infection, and severe hemorrhage which may result in hysterectomy (19). Moreover, women undergoing planned vaginal delivery are less likely to have severe morbidity or mortality compared to those delivered by CS on an emergency basis (19).

To minimize these surgical risks, it is important to identify new pragmatic treatment options that can decrease the incidence of CS in GDM patients. In this regard, the plausible clinical role of antenatal vitamin D supplementation in GDM patients is a novel area to explore, as suggested by recent vitamin D-related research. Existing studies suggest a possible association between vitamin D deficiency and GDM (20,21,22,23,24). Moreover, GDM prevalence tends to decrease on prenatal supplementation of vitamin D (25,26). Besides, maintaining the recommended optimum vitamin D status during pregnancy might be protective against CS, although the mechanism remains unclear (27,28,29). When vitamin D is complemented in GDM patients, it facilitates better glycemic control when measured by a decrease in fasting plasma glucose and/or insulin and improvement in homeostasis model of assessment-insulin resistance (20,21,22,23,24,30,31). All these vitamin D related findings in pregnancy and GDM formed the rationale for undertaking this study; to explore the risk of CS in (antenatal) vitamin D supplemented GDM patients.

## The intervention

Vitamin D is a fat-soluble hormone (32). It is available from diet and supplements in two physiologically inactive forms - D2 (ergocalciferol) and D3 (cholecalciferol) (33,34). Vitamin D3 is additionally synthesized in the skin on exposure to the sun (33). The active form of vitamin D, calcitriol 1,25-(dihydroxyvitamin) 2D, is produced on hydroxylation of vitamin D2 and D3 successively in the liver and kidneys (33,35). This active form plays a role in the physiology of pregnancy via the vitamin D receptors in uteroplacental tissue (33,35).

Recently, different clinical trials have tested the health effects of antenatal vitamin D supplementation in GDM patients. However, the route of vitamin D administration [parenteral (36) versus oral (37,38,39,40)], dosing, and the accompanying supplements (when used) varied among such trials. Some trials in pregnant women have used vitamin D as a sole supplement, (36,37,38) while others used it with co-supplements such as magnesium, zinc, or calcium (39,40). A trial that tested the role of intramuscular administration of vitamin D in GDM patients, used it as a single injection of 300.000 IU (36). In clinical trials that prescribed oral vitamin D, GDM patients were advised to take it at a dose of 50.000 IU, 2-3 weeks apart for 3-8 weeks (38,40). Other such trials asked GDM patients to take 200-500 IU of oral vitamin D twice daily for 6-16 weeks (37,41).

## What this review adds?

In GDM patients, the contemporary evidence of the effect of antenatal vitamin D supplementation on CS, and other obstetric outcomes are based on the evidence of clinical trials, like those reviewed in this paper. However, to the best of our knowledge, there has been no previous attempt to synthesize the overall rigor of such evidence by systematic review and meta-analysis. Therefore, this paper reviews this poorly evidenced area of GDM literature and synthesizes new evidence based on the existing highest quality of epidemiological studies (i.e., double-blinded randomized clinical trials). In addition, as this study involved GDM mothers who were not on insulin treatment, the latter’s therapeutic effects are unlikely to bias this findings of this study.

## Aim

This study aimed to compare the risk of CS between non-insulin treated GDM patients supplemented antenatally with vitamin D containing supplements and placebo. The auxiliary objective was to compare the risk of macrosomia, polyhydramnios, pre-eclampsia, and pre-term delivery among these treatment groups.

## Material and Methods

**Inclusion criteria:** 1. Study design: Parallel-arm (any number of arms) double-blinded randomized controlled clinical trials of any duration were eligible. 2. Participant: The eligible participants were adult (18 years or older) females diagnosed with GDM by American Diabetes Association criteria (42,43), between 24-28 weeks of their concurrent pregnancy who received the intervention of interest before initiation of insulin therapy. 3. Intervention compared: The above-described trials should compare the following interventions - vitamin D (in D2 or D3 form or both; as a sole supplement or adjunct to any other supplements) with placebo. Vitamin D supplementation was accepted irrespective of its dose and route of administration; oral or intramuscular. 4. Outcome: The trials must report the frequency of CS observed in each of the studied treatment groups, post-intervention.

**Exclusion criteria:** 1. Study design: Differing from that described in the inclusion criteria, which included observational study designs, single-arm interventional studies, and cross-over trials. 2. Participants: With diabetes of any other type except GDM or those diagnosed previously with GDM were excluded from this review.

The secondary outcomes of interest were the risk of macrosomia, polyhydramnios, pre-eclampsia, and pre-term delivery. However, these did not contribute to the inclusion criteria. This review follows the PRISMA (44) reporting guideline and does not have a pre-published protocol.

The search for eligible trials was conducted in electronic databases (PubMed, Embase, and Scopus) with no restriction to date or language. The following search strategy was used in PubMed: “vitamin D” or calciferol OR “vitamin D2” or ergocalciferol or “vitamin D3” or cholecalciferol or cholecalciferol (MeSH) or “ergocalciferols” (MeSH) AND “diabetes, gestational” (MeSH) and “gestational diabetes” or GDM. The search was restricted to clinical trials by using the filters “Clinical Trial” and “Randomized Controlled Trial.” Identical search terms were used for searching the other databases. The last date of database search was 07 February, 2020.

The papers identified by the electronic database search were skimmed for trials matching this review’s eligibility criteria. Publications were read in full text when they seemed to match these criteria or in circumstances where a decision of their inclusion or exclusion was not possible by reading the titles and abstracts only. Besides the above, an auxiliary search was conducted in the references of the papers that were included in this review.

Then the following data were extracted from the included trials: author information (first author’s last name and year of publication), study design (randomization, blinding, if placebo-controlled, single or multicentric, funding, ethical clearance, trial ID), participants (diagnosis, gestational age of GDM diagnosis, number randomized, mean age, participant consent, trial nation), interventions (intervention/s received by each of the trial arms), and outcomes. Using the appropriate tool from the Cochrane Collaboration, the risk of selection bias in the trials (based on random sequence generation and concealment of participant allocation), performance bias, detection bias, attrition bias, reporting bias and miscellaneous bias were assessed and categorized as high risk, low risk, and unclear risk (45).

The first author conducted the database search and retrieved the eligible trials and their data. The co-author subsequently rechecked it. The risk of bias in the respective trials was assessed by each author independently, and then the findings were cross referenced and matched. The authors resolved disputes in their opinion at all stages of this review by discussion.

The intervention effects on the outcomes were compared across the trials by the random-effect model meta-analysis (DerSimonian and Laird) method, and the summary effect was determined in risk ratios (RR). Despite the relative homogeneity of the participant characteristics and study design, a random-effect model was used since the vitamin D supplement adjuncts used between the trails were not identical. To determine the effects of vitamin D as a chief supplement, in trials that used it in multiple treatment arms, we chose one that included a fewer number of vitamin D adjuncts. For meta-analyses, when an outcome occurred in one of the intervention arms of a trial only, 0.5 was added to each cell of the 2x2 table. Heterogeneity was assessed using the p-value of Cochranes Q (statistical significance determined at p<0.1) in conjunction with I^2^ statistics (0-40%, 30-60%, 50-90%, and 75-100% represented less, moderate, substantial, and considerable heterogeneity, respectively) (45). Funnel plots were used to visually assess publication bias.

Finally, sensitivity analyses were performed, in which the meta-analysis for the respective outcomes was iterated using a fixed-effect model (inverse-variance method) and also by excluding a study each time (using both fixed-effect and random effect model). At p<0.05 and 95% confidence interval, results were considered statistically significant. The Stata statistical software (StataCorp, College Station, Texas, USA; version 16) was used to perform statistical analyses.

## Results

**Figure 1:**
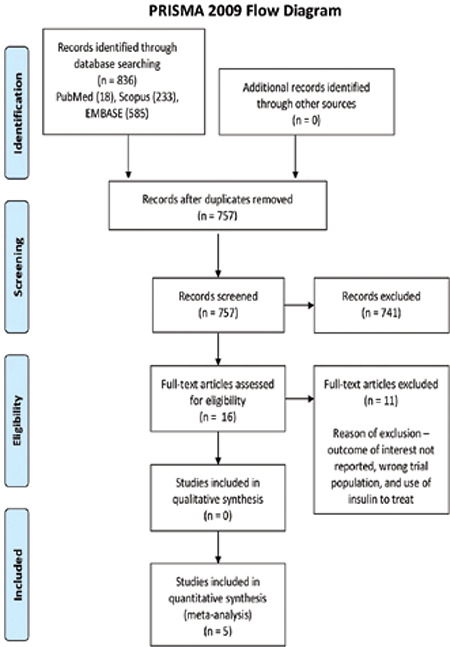
PRISMA 2009 Flow Diagram [From: Moher D, Liberati A, Tetzlaff J, Altman DG, The PRISMA Group (2009). Preferred Reporting Items for Systematic Reviews and Meta-Analyses: The PRISMA Statement. PLoS Med 6(7): e1000097. doi:10.1371/journal.pmed1000097]

The initial electronic search returned 836 citations. After excluding the duplicates, the titles and abstracts of 757 papers were read. For 16 studies, full-text reading ensued. Finally, five trials meeting the eligibility criteria of this review were included for the risk of bias assessment and quantitative analysis (Figure 1) (46,47,48,49,50). These trials were published between 2015-19, were primarily single centered (47,48,49,50,51) except one (46), and based on about 380 GDM patients from Iran. The average age of these participants was approximately between 28-32 years (46,47,48,49,50).

Two of these trials (48,50) tested vitamin D as a sole supplement in one of their treatment arms (48). In the intervention arms of the remaining trials, vitamin D was co-supplemented with another supplement including probiotics, magnesium, calcium, and zinc (46,47,49). All trials had a placebo arm (46,47,48,49,50). Each trial reported both the primary and secondary outcomes (46,47,48,49,50).

Regarding the appraisal of the studies, overall the trials are at a low risk of bias except for unclear risk of allocation concealment in four trials (46,47,49,50) and performance bias in one trial (47). Table 1 presents the salient features and the risk of bias assessment of the reviewed trials (46,47,48,49,50).

**Figure 2:**
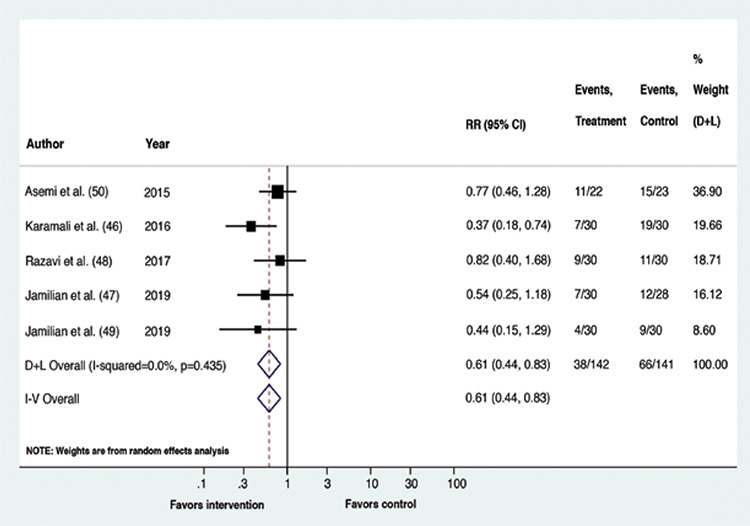
Forest plot: Comparison between vitamin D supplemented group and placebo for the outcome cesarean section

**Figure 3:**
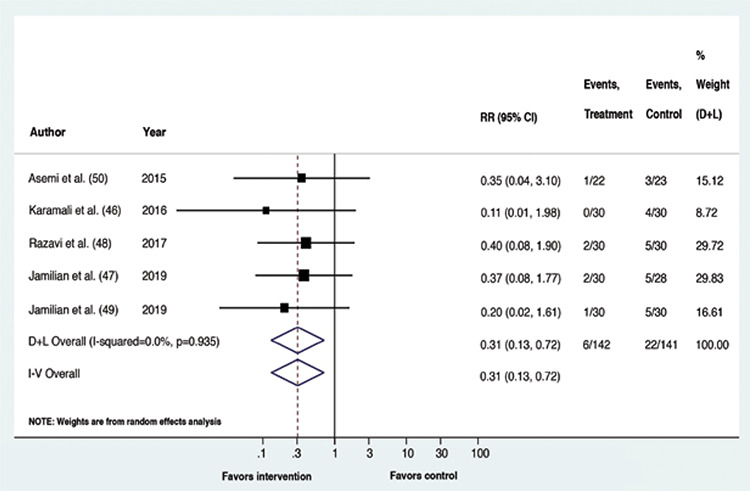
Forest plot: Comparison between vitamin D supplemented group and placebo for the outcome macrosomia

**Figure 4:**
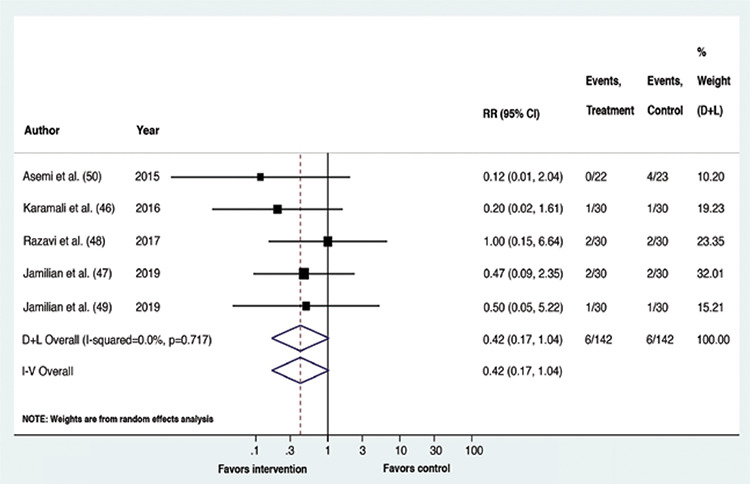
Forest plot: Comparison between vitamin D supplemented group and placebo for the outcome polyhydramnios

**Figure 5:**
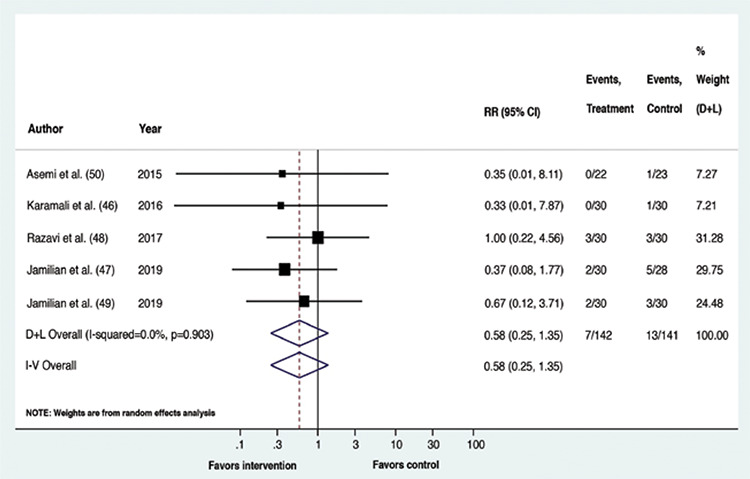
Forest plot: Comparison between vitamin D supplemented group and placebo for the outcome pre-eclampsia

**Figure 6:**
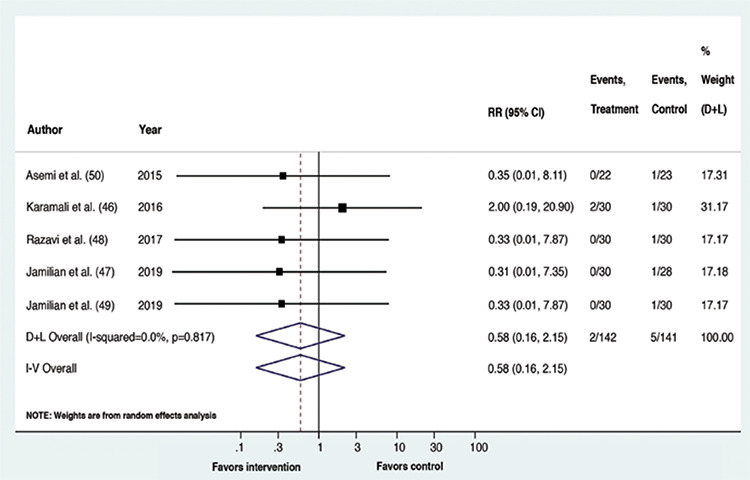
Forest plot: Comparison between vitamin D supplemented group and placebo for the outcome pre-term delivery

Upon meta-analysis, GDM patients receiving vitamin D containing supplements had a lower risk of experiencing CS (RR: 0.61, p=0.002, 95% confidence interval (CI): 0.44,0.83; I^2^=0%, p-value of Cochrane’s Q: 0.373) and macrosomia (RR: 0.31, p=0.006, 95% CI: 0.13,0.72; I^2^=0%, p-value of Cochrane’s Q: 0.935) than the placebo recipients. The risk of the remaining outcomes did not vary between the compared interventions. Overall, for all outcomes, statistical heterogeneity was classified as less, that is between 0-40% (45). The forest plots (Figure 2, 3, 4, 5, 6) depict the outcome data along with their effect sizes.

On visual inspection, the funnel plots (not shown) were not suggestive of any publication bias. Sensitivity analysis results were almost identical to the preliminary analyses (Table 2).

## Discussion

To summarize, five recent double-blinded randomized controlled Iranian trials (comprising about 380 GDM patients) compared the obstetric risk of CS, macrosomia, polyhydramnios, pre-eclampsia, and pre-term delivery between the prenatal recipients of vitamin D and placebo. The risk of bias in the trials was predominantly low with occasional unclear risk of bias components (46,47,48,49,50). The meta-analyses suggested that in GDM patients, antenatal vitamin D containing supplement recipients had a reduced risk of CS and macrosomia than those who took a placebo.

The evidence quality of CS and macrosomia was graded using the GRADE approach [GRADE Working Group (2004)] (52).Due to the unclear risk of bias present in some of the trials, the evidence was downgraded by one level to moderate-quality evidence.

The scope of contrasting the findings of this review with the existing literature is limited, due to its conceptual novelty. In this regard, there is a recent review by Cochrane collaboration comparing obstetric outcomes between the vitamin D (as a sole or complementary supplement) and placebo receiving pregnant females (27). It found no major difference in the risk of CS between these intervention groups (27). However, unlike this review, the Cochrane collaboration review (27) was not specific to the GDM subpopulation.

The implications of this review are discussed here. First, healthcare professionals caring for GDM patients might find this review of worth to expand their existing knowledge in this context. Next, research in this milieu may help to inform public health policy about endorsing prenatal vitamin D supplementation in GDM patients. The lower risks of macrosomia and CS due to vitamin D supplementation may encourage future researchers to investigate if there is a causal relationship between these. Moreover, future researchers from nations other than Iran may also consider researching this context to test if these paper’s findings are externally valid or not.

The following are the strengths of this review. First, this is perhaps the first systematic review that attempted to synthesize evidence in this study’s context. Second, the findings of this review are likely to be rigorous as it utilized evidence from double blinded randomized controlled trials, the highest level of epidemiological evidence. Third, this review is expected to be more comprehensive as its database search method was not restricted to any date or language. Lastly, the meta-analysis findings regarding CS and macrosomia are likely to be robust due to their similarity with the sensitivity analysis.

Despite these strengths, there are certain limitations of this paper. At the review level, the number of trials investigating the context was relatively few, which might have compromised the external validity of this meta-analysis. At the outcome level, by including intervention arms of trials that tested vitamin D along with other nutritional adjuncts, it is difficult to conclude if the observed effects were influenced by the latter. At the study level, the weaknesses were the unclear risk of bias (46,47,49,50), single centric study design (47,48,49,50), and relatively small sample size (46,47,48,49,50). Additionally, as all trials were Iran-based (46,47,48,49,50), the findings are unlikely to be generalizable to the global population.

## Conclusion

The contemporary evidence in non-insulin treated GDM patients from Iran suggests that antenatal vitamin D containing supplements decreases the risk of CS and macrosomia, compared to placebo. However, to increase the external validity of these findings, methodologically rigorous trials from different parts of the globe might be useful in the future. Furthermore, future trials may use vitamin D as the sole supplement to specifically identify its effects on obstetric outcomes in GDM patients.

## Figures and Tables

**Table 1:**
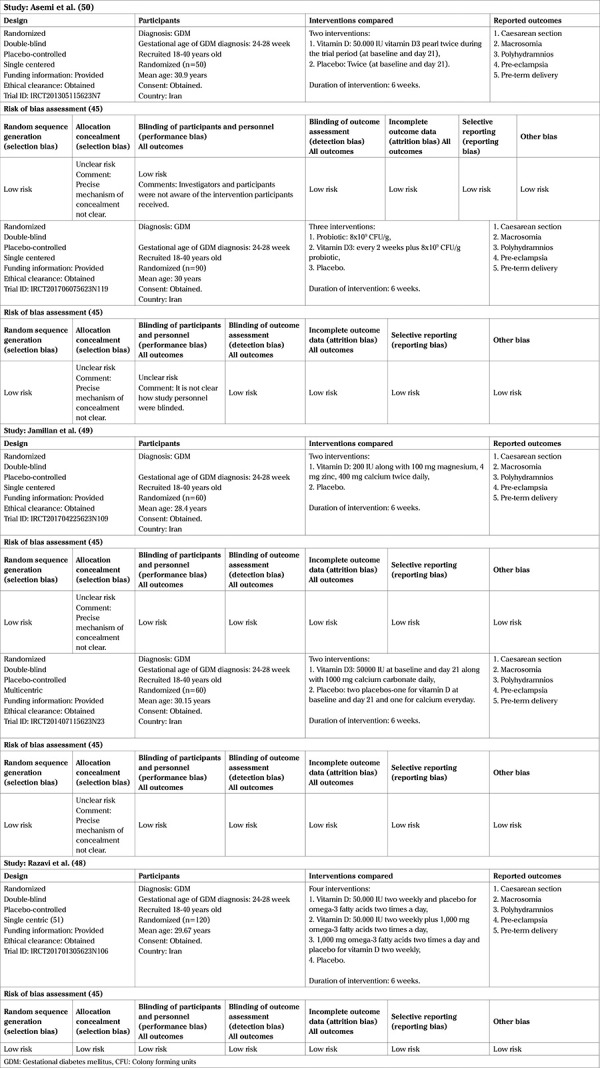
Salient features of reviewed papers and risk of bias assessment

**Table 2:**
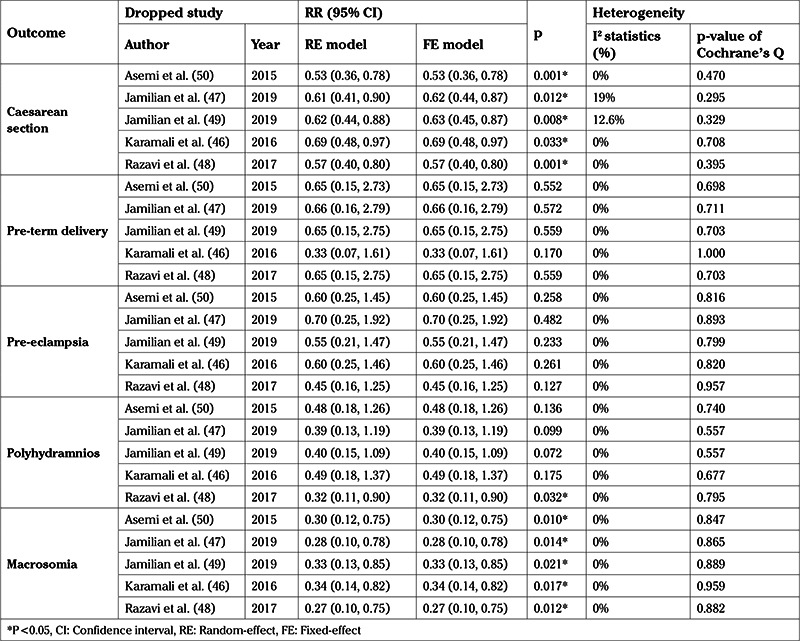
Sensitivity analysis (by dropping a trial in each meta-analytic iteration)
